# Giant caseous mitral annular calcification mimicking ventricular pseudoaneurysm

**DOI:** 10.1111/jocs.15509

**Published:** 2021-03-19

**Authors:** Nicola Galea, Giacomo Pambianchi, Francesco Cilia, Giuseppe Mancuso, Livia Marchitelli

**Affiliations:** ^1^ Department of Radiological, Oncological, and Pathological Sciences Sapienza University of Rome Rome Italy; ^2^ Department of Experimental Medicine Sapienza University of Rome Rome Italy

**Keywords:** caseous mitral annular calcification, cardiac computed tomography, heart valve diseases, mitral valve, pseudoaneurysm

## Abstract

An 82‐year‐old woman with precordial pain at rest was admitted to the Emergency Department for possible cardiac heart disease; electrocardiogram excluded ischemia and high‐sensitive troponin was normal. Echocardiogram revealed a hyperechoic mass adjacent to the mitral annulus. Electrocardiography‐gated computed tomography (CT) angiography exam confirmed the presence of the mass protruding into the atrioventricular groove, adjacent to the posterior mitral. On the precontrast images the lesion was hyperdense with some scattered central calcific spots. CT findings are typical of a giant caseous calcification of the mitral annulus and excluded the diagnoses of pseudoaneurysm (it does not show any communication with the left ventricular cavity), neoplasm/abscess (complete caseous/calcified content) or infected/abscessified mitral calcification (absence of internal hypodense core). This is a benign condition that can be easily misdiagnosed as ventricular aneurysm or pseudoaneurysm on the contrast‐enhanced images, when the caseous content is isodense to the iodinated blood pool.

## 1 CLINICAL SCENARIO

An 82‐year‐old woman presented to the Emergency Department with acute precordial pain and blurry vision.

Electrocardiogram (ECG) and high‐sensitive troponin were normal and excluded myocardial infarction.

Echocardiography detected a large, heterogeneous, hyperechoic mass adjacent to the mitral annulus.

## 2 COMPUTED TOMOGRAPHY (CT) FINDINGS

**Figure 1:**
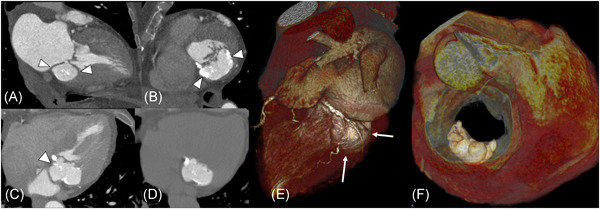
Contrast‐enhanced CT images (A–C) showed a 50 × 43 mm hyperdense crescent‐shaped mass located within a bi‐lamination of the posterior mitral annulus(arrowheads) in a 82‐year‐old woman. Noncontrast CT images (D) excluded diagnosis of pseudoaneurysm, by revealing homogeneous caseous content with some calcifications. On 3D‐volume‐rendered images the lesion mimics ventricular outpunching (E) and appears nonobstructing trans‐mitral flow (F). CT, computed tomography

ECG‐gated computed tomography angiography examination confirmed a well‐marginated, lobulated, 5 × 4 cm hyperdense mass, protruding into the atrioventricular groove (Figure 1A, arrowheads), adjacent to the posterior mitral leaflet (Figure 1B,C, arrowheads).

On the unenhanced scan, the lesion was hyperdense with some scattered central calcific spots (Figure 1D).

On 3D‐volume‐rendered images the lesion mimicked a ventricular outpunching (Figure 1E, arrows) without obstruction of mitral valve opening during diastole (Figure 1F).

The lesion was surrounded by a thin hypodense layer as it seems to originate within a bilamination of the posterior mitral annulus.

The adjacent ventricular wall showed normal thickness and density, and no signs of soft tissue/fat stranding was noted nearby the lesion.

The aforementioned CT findings are typical of a giant caseous calcification of the mitral annulus (CCMA),^1^ and excludes the diagnoses of pseudoaneurysm (it does not show any communication with the left ventricular cavity),^2^ neoplasm/abscess (complete caseous/calcified content) or infected/abscessified mitral calcification (absence of internal hypodense core).^3^

## 3 CONCLUSION

CCMA is a rare evolution (around 0.68%) of a calcified mitral annulus due to caseous transformation of the inner material.^1^, ^3^ In CCMA, a peripheral rim surrounds the caseous material, composed of a mixture of calcium, fatty acids, and cholesterol, and gives the characteristic hyperdensity on noncontrast CT images. This is a benign condition that can be easily misdiagnosed as ventricular aneurysm or pseudoaneurysm on the contrast‐enhanced images, when the caseous content is isodense to the iodinated blood pool.

Since cardiac‐CT is frequently required to clarify equivocal cases, it should include the noncontrast scan when employed in mass characterization.

## CONFLICT OF INTERESTS

The authors declare that there are no conflict of interests.

## ETHICS STATEMENT

This manuscript and all of its content meet the ethical guidelines, including adherence to the legal requirements of the study country. Informed Consent for the publication was obtained by the patient.
